# Unlocking the potential of antibody-drug conjugates in cervical cancer: emerging targets and clinical trials

**DOI:** 10.3389/fphar.2025.1636120

**Published:** 2025-07-08

**Authors:** Yue Zhang, Yao Ding, Yiran Liao, Jin Shu, Yi Gong

**Affiliations:** ^1^ Phase I Clinical Trial Center, Chongqing University Cancer Hospital, Chongqing, China; ^2^ Chongqing Key Laboratory of Translational Research for Cancer Metastasis and Individualized Treatment, Chongqing University Cancer Hospital, Chongqing, China

**Keywords:** cervical cancer, antibody-drug conjugate, tumor-associated antigen, target, clinical trial

## Abstract

Despite significant advances in immune checkpoint inhibitors and targeted therapies, treatment options remain limited for recurrent and metastatic cervical cancer (r/mCC) following progression on first-line therapy. There persists a substantial unmet clinical need for novel therapeutic strategies that are both effective and well-tolerated. In recent years, antibody-drug conjugate (ADC) have gained increasing attention as an emerging form of precision chemotherapy with targeted delivery capabilities, offering a promising therapeutic approach for r/mCC. With the approval of tisotumab vedotin (TV), a tissue factor (TF)-targeting ADC, for the treatment of r/mCC, an increasing number of ADCs targeting different antigens have demonstrated highly encouraging therapeutic potential in cervical cancer patients. The identification of ideal antigenic epitopes represents a critical factor in ADC development. This review outlines promising tumor-associated antigens (TAAs) for ADC targeting in cervical cancer and their biological functions, such as human epidermal growth factor receptor 2 (HER2), trophoblast cell surface antigen 2 (Trop-2), mesothelin, nectin cell adhesion molecule 4 (Nectin-4). We also summarize the clinical applications and research progress of corresponding ADC, and provide novel perspectives for future ADC development and clinical research strategies.

## 1 Introduction

Cervical cancer is the second most common malignant tumor affecting women’s health globally, with over 660,000 new cases and more than 340,000 deaths reported annually worldwide (Bray et al., 2024). The primary treatment for cervical cancer patients typically involves surgery, chemoradiotherapy, or a combination of these modalities, with the choice depending on the stage of the cancer. In recent years, the incorporation of pembrolizumab, an anti-programmed death 1 (PD-1) monoclonal antibody (mAb), and bevacizumab, a humanized anti-vascular endothelial growth factor (VEGF) mAb, has significantly transformed the therapeutic paradigm for cervical cancer.

The phase III GOG-240 trial (NCT00803062) showed that adding bevacizumab to chemotherapy significantly improved median overall survival (OS) by 3.7 months (17.0 months vs. 13.3 months) in patients with recurrent, persistent, or metastatic cervical cancer (Tewari et al., 2014). Combination chemotherapy plus bevacizumab has become the standard first-line (1L) treatment for patients with advanced cervical cancer. Based on the phase III KEYNOTE-826 trial (NCT03635567) results, pembrolizumab in combination with chemotherapy (with or without bevacizumab) is the preferred 1L treatment for programmed death-ligand 1 (PD-L1)-positive recurrent and metastatic cervical cancer (r/mCC) (Colombo et al., 2021). Treatment options after 1L progression remain limited, with second-line (2L) and subsequent chemotherapy regimens typically showing low response rates and median progression-free survival (PFS) of 3–6 months (Porras et al., 2018; Boussios et al., 2016). Patients with metastatic, recurrent, or persistent cervical cancer face poor prognosis, demonstrating a 5-year survival rate of less than 20% (Giudice et al., 2021). There remains a substantial unmet need for novel treatment strategies that are both effective and well-tolerated among the population with r/mCC, who continue to face poor prognoses and limited therapeutic options.

The rapid advancement of targeted therapy has brought renewed hope to these patients. Targeted therapy relies on the overexpression of specific antigens on the surface of tumor cells, which are rarely or not expressed in normal cells. In-depth investigation of tumor-associated antigens (TAAs) provides crucial insights into the pathological mechanisms of epithelial barrier dysfunction and facilitates the development of precision therapies, demonstrating significant translational value particularly in the field of targeted cancer treatment. A variety of targeted therapeutics have been developed to address different TAAs, including mAbs, bispecific antibodies, small molecule inhibitors, antibody-drug conjugates (ADCs), nanoparticles, and chimeric antigen receptor T cells. Among these, ADCs have emerged as a revolutionary targeted treatment approach for both hematologic malignancies and various refractory and advanced solid tumors.

ADC is a targeted biologic agent that links a cytotoxic drug (known as the payload) to a mAb via a chemical linker (Chau et al., 2019). By utilizing the mAb as a carrier, ADCs effectively deliver small-molecule cytotoxic drugs to target tumor cells (Tarantino et al., 2022). This approach preserves the antitumor properties of the cytotoxic payload while reducing its off-target effects, thereby significantly improving the benefit-risk ratio of anticancer therapy. The ability of ADCs to selectively target and eradicate cancer cells has markedly advanced the treatment of difficult-to-treat malignancies. Preclinical and clinical research on ADCs for the treatment of cervical cancer is being actively conducted worldwide. These ADCs offer additional therapeutic options for patients with advanced cervical cancer, particularly for those who have progressed after multiple lines of therapy, and hold the potential to improve clinical outcomes. This article reviews potential ADC targets in cervical cancer and their mechanisms of action in tumor biology, summarizes the latest clinical trial results of representative ADCs (as shown in Table 1) in cervical cancer, and provides an outlook on the future prospects and directions of ADC applications.

**TABLE 1:** Clinical trials of ADCs in cervical cancer*.*

Target	ADC	Antibody	Payload	Linker	Clinical state	Indications	Main trial	Phase	ClinicalTrials gov identifiers
TF	TV	IgG1	MMAE	Valine-citruline cleavable linker	Approved	Recurrent and metastatic cervical cancer with disease progression on or after chemotherapy	InnovaTV 204 InnovaTV 301 InnovaTV 205	II III I/II	NCT03438396 NCT04697628 NCT03786081
	MRG004A	IgG1	MMAE	Valine-citruline cleavable linker	In research	Solid tumors	MRG004A-001	I/II	NCT03941574
	XB002	Clone 25A3	ZymeLink Auristatin	Zovodotin	In research	Solid tumors	JEWEL-101	I/II	NCT04925284
HER2	T-DXd	Trastuzumab	DXd	Maleimide glycine-phenylalanine-glycine peptide	Approved	Unresectable or metastatic HER2-positive solid tumors	DESTINY-PanTumor02 DESTINY-PanTumor01	II II	NCT04482309 NCT04639219
	RC48	Hertuzumab	MMAE	Valine-citruline cleavable linker	Approved	HER2-overexpressing locally advanced or metastatic GC/GEJC and metastatic UC	RC48-C018 RC48-C030	II II	NCT04965519 NCT06155396
	IBI354	Trastuzumab	Camptothecin derivative	Undisclosed	In research	Solid tumors	CIBI354A101	I/II	NCT05636215
Trop-2	SG	IgG1	SN-38	CLA2	Approved	Metastatic TNBC,HR+/HER2- BC and UC	EVER-132–003	II	NCT05119907
	SKB264	IgG1	KL610023	2-methylsulfonyl pyrimidine	Approved	Locally advanced or metastatic TNBC and EGFR mutation-positive non-squamous NSCLC after 2L systemic therapy	SKB264-II-06 MK-2870–020	II III	NCT05642780 NCT06459180
Mesothelin	RC88	IgG1	MMAE	C75:Py-MAA-Val-Cit-PAB	In research	Solid tumors	RC88-C001	I/II	NCT04175847
	AR	MF-T	DM4	Disulfide linker	In research	Solid tumors	—	I	NCT01439152
Nectin-4	9MW-2821	IgG1	MMAE	Valine-citruline cleavable linker	In research	Solid tumors	CTR20220106	I/IIa	NCT05216965
	ADRX-0706	IgG1κ	AP052	Undisclosed	In research	Solid tumors	ADRX-0706–001	I	NCT06036121

Abbreviations: ADCs, antibody-drug conjugates; TF, tissue factor; TV, tisotumab vedotin; MMAE, monomethyl auristatin E; HER2, human epidermal growth factor receptor 2; GC/GEJC, gastric/gastroesophageal junction cancer; UC, urothelial carcinoma; Trop-2, trophoblast cell surface antigen 2; SG, sacituzumab govitecan; TNBC, triple-negative breast cancer; HR, hormone receptor; BC, breast cancer; EGFR, epidermal growth factor receptor; NSCLC, non-small cell lung cancer; 2L, second-line; AR, anetumab ravtansine; Nectin-4, nectin cell adhesion molecule 4.

## 2 Tissue factor (TF)

TF, also known as coagulation factor III, thromboplastin, F3, or CD142, is a transmembrane glycoprotein is encoded by the F3 gene located on chromosome 1p21.3 in humans, spanning approximately 12.4 kb in length (Platform MCPP and monoclonal Abs A-T, 2025). Under physiological conditions, TF binds the coagulation serine protease factor VII/VIIa (FVII/VIIa) to initiate the extrinsic coagulation pathway for hemostasis (Mackman, 2004). TF shows basal expression in vascular cells (e.g., smooth muscle cells, fibroblasts, and pericytes) but is aberrantly overexpressed in cervical cancer and other solid tumors (Leppert and Eisenreich, 2015). TF overexpression is associated with increased tumor aggressiveness and poor prognosis, and plays a role in tumor progression, invasion, metastasis, and angiogenesis.

The most critical component of TF’s non-hemostatic functions is the direct or indirect cellular signaling induced by the TF-FVIIa complex. On the surface of tumor cells, the TF-FVIIa complex generates pro-angiogenic factors, such as VEGF, through protease-activated receptor 2 (PAR2)-mediated intracellular signaling pathways, thereby stimulating tumor angiogenesis (Kocatürk and Versteeg, 2013)^,^ (Schaffner and Ruf, 2009). Additionally, it activates the janus kinase 2 and signal transducers and activators of transcripition 5 (JAK2-STAT5) pathway to produce the anti-apoptotic protein B-cell lymphoma 2 (Bcl-2), which inhibits cancer cell apoptosis (Fang et al., 2008). PAR2 stabilizes β-catenin, leading to tumor cell invasion. PAR2 also activates the mitogen-activated protein kinase (MAPK) and extracellular signal-regulated kinase (ERK) 1/2 pathways, while upregulating β-arrestin. This phosphorylates cofilin, triggering actin filament polymerization at the leading edges of invading tumor cells, thereby enhancing their invasive and metastatic potential (Ahmadi et al., 2023). TF can stimulate tumor formation by regulating immunity and promoting inflammation (Hisada and Mackman, 2019).

The expression of TF by tumors may promote metastasis by inducing fibrin encapsulation of tumor cells, thereby trapping them within microvessels. Additionally, intravascular thrombosis may activate endothelial cells and induce the expression of adhesion molecules, facilitating tumor cell extravasation into extravascular spaces (Kasthuri et al., 2009). Moreover, TF can induce the secretion of matrix metalloproteinases, which degrade the surrounding extracellular matrix, thereby facilitating tumor cell invasion into adjacent tissues and promoting distant metastasis (Eble and Niland, 2019). TF is highly expressed in up to 95% of cervical cancer (Zhao et al., 2018), and has been identified as a promising therapeutic target for cervical cancer.

### 2.1 ADCs targeting TF

**FIGURE 1:**
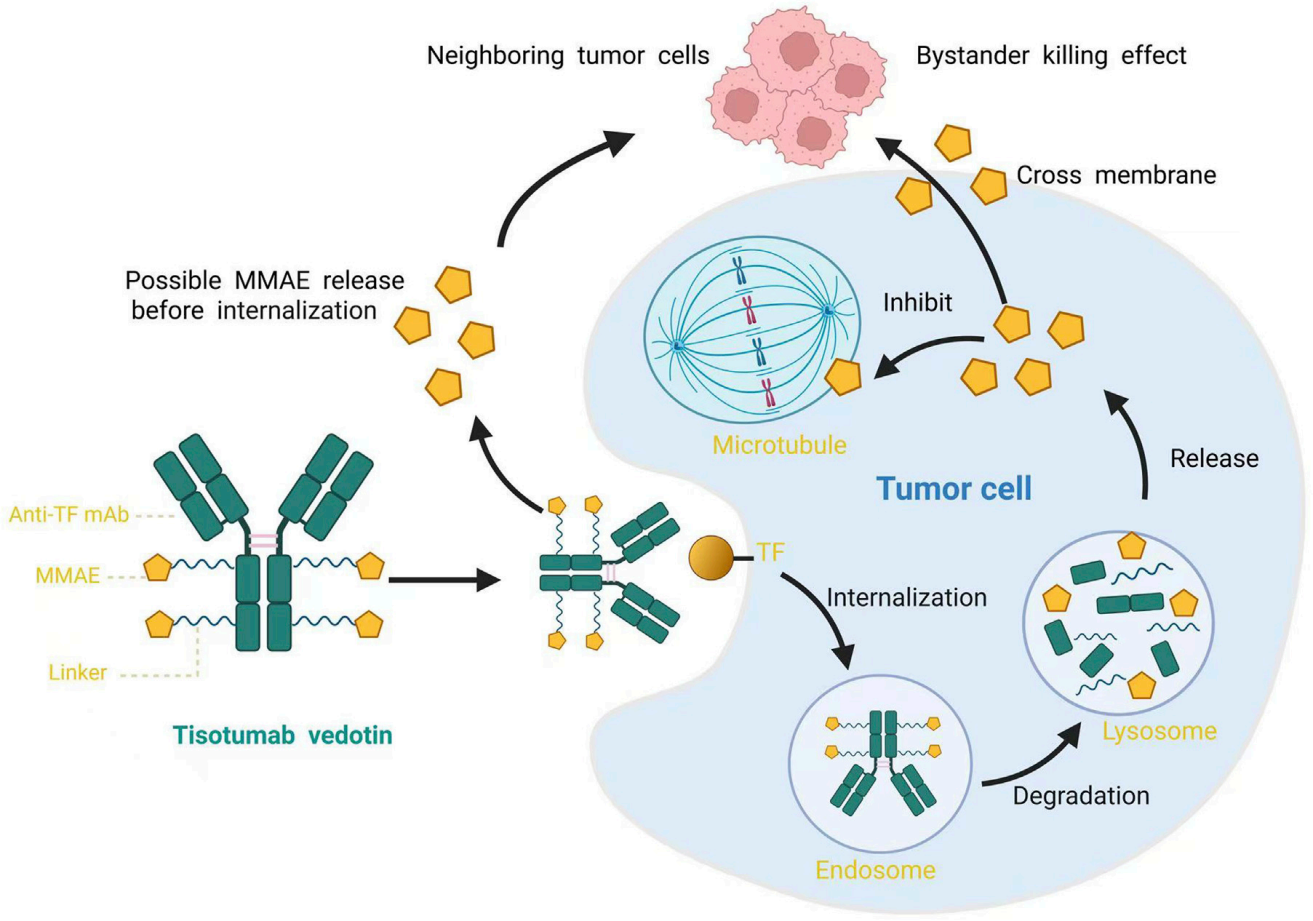
Schematic diagram of Tisotumab Vedotin structure and its mechanism of action. TF, tissue factor; MMAE, monomethyl auristatin E.

Tisotumab vedotin (TV, Tivdak^®^), the first ADC approved for the treatment of cervical cancer, consists of a human TF-specific mAb, a protease-cleavable linker, and the highly potent cytotoxic payload monomethyl auristatin E (MMAE)—a microtubule-disrupting agent (Breij et al., 2014), as shown in Figure 1. TV initially binds to TF on the cell surface and is internalized. The released MMAE inhibits tubulin polymerization, disrupting microtubule dynamics. This triggers mitotic arrest and subsequent apoptosis of target cells. It exerts direct cytotoxicity and induces a “bystander killing effect” on neighboring cells (Hong et al., 2020). Secondly, TV effectively engages immune cells and drives tumor cell death via Fcγ receptor-mediated mechanisms such as antibody-dependent cellular phagocytosis and antibody-dependent cellular cytotoxicity. Additionally, TV can suppress the TF-mediated signaling pathway triggered by FVIIa, thereby further augmenting its anti-tumor efficacy (Breij et al., 2014).

The innovaTV 204 trial (NCT03438396), a phase II single-arm study, assessed the efficacy of TV in 102 patients with r/mCC (Coleman et al., 2021). Results demonstrated an objective response rate (ORR) of 24%, including a 7% complete response (CR) and 17% partial response (PR). The disease control rate (DCR) was 72%, with 79% of patients experiencing tumor shrinkage from baseline. The median time to response was 8.0%, and the median duration of response (DoR) was 8.3 months—surpassing the typical 2–6 months observed with single-agent chemotherapy. Additionally, the median PFS and OS were 4.2 and 12.1 months, respectively, as shown in Table 2. Based on the results, the U.S. Food and Drug Administration (FDA) accelerated approval of TV in September 2021 for r/mCC with disease progression on or after chemotherapy (Heitz et al., 2023). The most common treatment-related adverse events (TRAEs) of TV included alopecia, epistaxis, nausea, conjunctivitis, fatigue, and dry eye. Grade 3 or worse TRAEs were neutropenia, fatigue, ulcerative keratitis, and peripheral neuropathies (Coleman et al., 2021). The FDA’s black box warns of eye adverse events with TV, recommending close monitoring of eye conditions during treatment and the use of eye care and corticosteroid eye drops.

**TABLE 2:** Efficacy outcomes of ADCs from clinical trials in cervical cancer patients.

ADC	Target	Trial	ORR (%)	DCR (%)	mDoR (months)	mPFS(months)	mOS(months)
TV	TF	InnovaTV 204	24	72	8.3	4.2	12.1
T-DXd	HER2	DESTINY-PanTumor02	50	67.5	14.2	7.0	13.6
RC48	HER2	RC48-C018	31.8	86.4	5.52	4.37	unmature
SG	Trop-2	EVER-132–003	50	94	9.2	8.1	—
RC88	Mesothelin	RC88-C001	35.3	—	—	—	—
9MW-2821	Nectin-4	CTR20220106	32.1	81.1	6.3	3.9	16.0

Abbreviations: ADCs, antibody-drug conjugates; ORR, objective response rate; DCR, disease control rate; mDoR, median duration of response; mPFS, median progression-free survival; mOS, median overall survival; TV, tisotumab vedotin; TF, tissue factor; HER2, human epidermal growth factor receptor 2; SG, sacituzumab govitecan; Trop-2, trophoblast cell surface antigen 2; Nectin-4, nectin cell adhesion molecule 4;/, not available.

The innovaTV 205 (NCT03786081) is a global, multicenter, open phase I/II clinical study (Vergote et al., 2023a). The dose-expansion arms evaluated the antitumor activity and safety of TV in combination with carboplatin as therapy (arm D) or in combination with pembrolizumab as 1L (arm E) or 2L/third-line (3L) (arm F) therapy for r/mCC. The interim results of all three arms showed encouraging and persistent antitumor activity, with ORR of 54.5%, 40.6% and 35.3% in arms D, E and F, respectively. The median DoR was 8.6 months, not reached, and 14.1 months, in arms D, E, and F, respectively. The results from innovaTV 205 could reshape the future treatment landscape for r/mCC.

The innovaTV 301 (NCT04697628) was a global, randomized, open-label phase III study that evaluated the efficacy and safety of TV versus investigator’s choice chemotherapy (topotecan, vinorelbine, gemcitabine, irinotecan, or pemetrexed) in 502 patients with r/mCC who had previously received standard of care chemotherapy doublet ± bevacizumab ± anti-PD-(L)1 therapy (Vergote et al., 2023b). Compared to chemotherapy, the TV arm showed a 30% lower risk of death, along with a significantly longer median OS (11.5 months vs 9.5 months). PFS was also superior in the TV arm. The confirmed ORR were 17.8% for TV and 5.2% for chemotherapy, respectively. The National Comprehensive Cancer Network (NCCN) Guidelines recommend TV as a preferred therapy option for the treatment of r/mCC with disease progression on or after chemotherapy regardless of biomarker status (Abu-Rustum et al., 2023). TV may become the new standard of r/mCC after 1L systemic therapy.

MRG004A is a novel ADC that utilizes a glycosite-specific conjugation technology to link an anti-TF mAb with MMAE. As of May 2024, preliminary safety and efficacy data from phase I/II first-in-human study of MRG004A (NCT03941574) in patients with solid tumors indicated that in 2 patients with cervical cancer with four prior therapy lines, 1PR and 1 SD (Park et al., 2024). This study enrolled only a limited number of cervical cancer patients. Notably, MRG004A has also demonstrated encouraging antitumor activity in solid tumors including pancreatic cancer. The agent received FDA Orphan Drug Designation for pancreatic cancer in December 2023, followed by Fast Track designation in March 2024. Common TRAEs of MRG004A include conjunctivitis, anemia, and hypoalbuminemia.

XB002 is an anti-TF ADC that consists of an anti-TF mAb (clone 25A3) with high affinity to TF conjugated at the cysteine residues to the protease-cleavable drug-linker zovodotin. The advantage of this drug over TV and MRG004A is that its monoclonal antibodies do not disrupt the clotting cascade, minimizing the risk of bleeding (Kantak et al., 2024). Currently, XB002 is being evaluated in a first-in-human clinical study (NCT04925284), which includes cervical cancer patients with ≤2L in the tumor-specific cohort. While no safety or efficacy data have been reported in patients yet, the compound demonstrated complete tumor regression in murine cervical cancer patient-derived xenograft models within 30 days of treatment (Kantak et al., 2024). Collectively, these TF-ADCs demonstrate strong therapeutic potential for cervical cancer treatment.

## 3 Human epidermal growth factor receptor 2 (HER2)

HER2 is a transmembrane protein encoded by the erbB2 gene located on chromosome 17q12 (Popescu et al., 1989). As a member of the HER/erbB family, it shares high homology with HER1 (epidermal growth factor receptor, EGFR), HER3, and HER4. These receptors regulate key signaling pathways for cell growth, differentiation, and survival (Popescu et al., 1989; Rubin and Yarden, 2001). HER2 typically responds to extracellular signals by forming heterodimers with other erbB family members, thereby initiating intracellular signaling networks (Arkhipov et al., 2013). Heterodimers containing HER2 generate significantly stronger intracellular signals than other HER family pairings. Under normal physiological conditions, HER2 exhibits minimal surface expression in cells, resulting in fewer heterodimers and consequently weaker, more tightly regulated growth signals. However, HER2 gene amplification or protein overexpression leads to dramatically increased HER2 surface density. This overexpression promotes excessive homodimer (HER2/HER2) or heterodimer (e.g., HER2/HER3, HER2/HER4) formation. Notably, these dimers exhibit ligand-independent activation and demonstrate enhanced signaling potency, causing sustained activation of downstream pathways, particularly phosphatidylinositol-3 kinase/AKT serine/threonine tinase (PI3K/AKT) and MAPK pathway (Rubin and Yarden, 2001). This aberrant signaling drives uncontrolled cellular processes including proliferation, survival, invasion, and anti-apoptotic activity.

Additionally, abnormal HER2 signaling can increase the invasiveness and metastatic potential of tumor cells and activate multiple anti-apoptotic pathways, rendering tumor cells resistant to treatment (Spector and Blackwell, 2009; Citri and Yarden, 2006; Gutierrez and Schiff, 2011). Given its frequent overexpression in human malignancies contrasted with low expression in normal tissues, HER2 has emerged as an attractive therapeutic target for solid tumors. A systematic review and meta-analysis estimated the overall prevalence of HER2 overexpression in cervical cancer to be 5.7% and HER2 amplification to be 1.2% (Itkin et al., 2021). The NCCN Guidelines Panel recommends HER2 immunohistochemistry (IHC) testing for patients with advanced and r/mCC (Category 2A) (Abu-Rustum et al., 2023). Several HER2-targeting ADCs with novel payloads have demonstrated efficacy in cervical cancer.

### 3.1 ADCs targeting HER2

Fam-trastuzumab deruxtecan-nxki (T-DXd, DS-8201a, Enhertu^®^, Daiichi Sankyo) is an ADC composed of trastuzumab—a humanized anti-HER2 IgG1 mAb—linked to a topoisomerase I inhibitor (DXd) via a cleavable tetrapeptide-based linker (Ogitani et al., 2016). This linker design enhances stability during systemic circulation while minimizing systemic toxicity (Ogitani et al., 2016). The high membrane permeability of DXd enables potent bystander killing of HER2-low-expressing cells, representing a key advantage of T-DXd over conventional monoclonal antibodies (Suzuki et al., 2021). T-DXd has been approved for the treatment of metastatic HER2-positive or HER2-low breast cancer (BC), HER2-mutant non-small cell lung cancer (NSCLC), metastatic HER2-positive gastric/gastroesophageal junction cancer (GC/GEJC).

The DESTINY-PanTumor02 phase II trial (NCT04482309) evaluated T-DXd (5.4 mg/kg once every 3 weeks) in patients across seven cohorts with HER2-expressing (IHC 3+/2+ by local or central testing) locally advanced or metastatic solid tumors (Meric-Bernstam et al., 2024). Eligible patients had received ≥1L systemic treatment or had no alternative treatment options. In the cervical cancer cohort (including 40 patients), the ORR was 50.0% overall, while in the HER2 IHC 3+ subgroup, the ORR was 75.0%. The DCR was 67.5% and the median DoR was 14.2 months. The median PFS and OS in this cohort were 7.0 and 13.6 months in all patients (Meric-Bernstam et al., 2024).

In another open-label, multicenter, phase II DESTINY-PanTumor01 study (NCT04639219), patients with solid tumors harboring specific HER2-activating mutations were treated with T-DXd, demonstrating an ORR of 66.7% in cervical cancer patients (Li et al., 2023). Based on these data, Version 1.2024 of the NCCN Guidelines include T-DXd as a category 2A, useful in certain circumstances, 2L/subsequent therapy option for HER2-positive cervical cancer (IHC 3+/2+) (Abu-Rustum et al., 2023). On 5 April 2024, the FDA approved T-DXd for adult patients with unresectable or metastatic HER2-positive (IHC3+) solid tumors who have previously received systemic therapy and have no satisfactory alternative treatment options. The most common TRAEs included nausea, anemia, diarrhea, vomiting, and fatigue. The most common grade 3 or higher drug-related adverse events are neutropenia and anemia (Meric-Bernstam et al., 2024). A black box on the product label warns of the risk of interstitial lung disease and embryo-fetal toxicity.

Disitamab vedotin (DV, RC48, Aidixi^®^) is an ADC comprising the novel humanized anti-HER2 antibody Hertuzumab via a cleavable linker to the cytotoxic agent MMAE. Hertuzumab demonstrates higher HER2-binding affinity and stronger in vitro antibody-dependent cellular cytotoxicity activity compared to trastuzumab (Xu et al., 2021). RC48 have received approval in China for the treatment of patients with HER2-overexpressing (defined as IHC2+ or 3+) locally advanced or metastatic GC/GEJC and metastatic urothelial carcinoma (UC). A Phase II study (NCT04965519) evaluating the efficacy and safety of RC48 in HER2-expressing 2L r/mCC reported preliminary results in 22 evaluable patients, showing a confirmed ORR of 31.8%, median DoR of 5.52 months, DCR of 86.4%, and median PFS of 4.37 months. The median OS was unmature (Yuan et al., 2024). The most common TRAEs included alanine aminotransferase increased, aspartate aminotransferase increased, and white blood cell (WBC) count decreased. A phase II, single-arm, multicenter, open-label clinical trial (NCT06155396) is currently underway to evaluate the efficacy and safety of RC48 combined with zimberelimab (a PD-1 mAb) in patients with HER2-positive (IHC 2+/3+) r/mCC who have progressed after at least 1L platinum-based systemic therapy. The study is expected to conclude in February 2027. RC48 is a promising new drug for the treatment of HER2-overexpressed cervical cancer.

IBI354 is an ADC consisting of trastuzumab conjugated to a topoisomerase I inhibitor. A Phase I study of IBI354 (NCT05636215) in patients with advanced gynecologic cancer reported outcomes in 14 patients with HER2 2+/3+ cervical cancer and endometrial cancer. The ORR and DCR were 57.1% and 92.9%, respectively, including one endometrial cancer patient who achieved CR and 7 patients (4 with cervical cancer and 3 with endometrial cancer) who achieved PR (Shu et al., 2024). Most common TRAEs were anemia, leukopenia, nausea, and neutropenia.

## 4 Trophoblast cell surface antigen 2 (Trop-2)

Trop-2, also known as tumor-associated calcium signal transducer 2, epithelial glycoprotein-1, gastrointestinal antigen 733–1, and membrane component 1 surface marker 1, is a type I transmembrane glycoprotein and transmembrane calcium signal transducer encoded by the 1p32.1 locus on the short arm of chromosome 1 (Lipinski et al., 1981; Toumi and Mathe, 2023). The protein is frequently overexpressed in various epithelial malignancies (e.g., BC, colorectal carcinoma) while being virtually undetectable in normal adult tissues (Lipinski et al., 1981; Goldenberg et al., 2018). Studies have confirmed that Trop-2 plays a significant role in cell proliferation, apoptosis, cell adhesion, epithelial-mesenchymal transition, as well as tumorigenesis and progression. Trop-2 is typically expressed in cervical cancer, particularly in squamous cell carcinoma, but is not expressed in normal cervical tissues (Zeybek et al., 2020; Chiba et al., 2024; Varughese et al., 2011). Its overexpression in cervical cancer tissues is associated with International Federation of Gynecology and Obstetrics staging, histological grade, lymph node metastasis, depth of stromal invasion, and high expression of Ki-67 (Liu et al., 2013). Cervical cancer patients with positive Trop-2 expression exhibit poorer OS and PFS (Liu et al., 2013).

The biological role of Trop-2 in cervical cancer is still under investigation. High levels of Trop-2 protien can enhance the activity of the transcription factor activator protein 1 (AP-1) through the MAPK/ERK signaling pathway (Cubas et al., 2010; Lin et al., 2012). AP-1 promotes angiogenesis through VEGF, drives cell proliferation via cyclins and cyclin-dependent kinases (CDKs), induces apoptosis through Bcl-2 or Fas ligand, and mediates cell invasion and metastasis through matrix metalloproteinases (Shvartsur and Bonavida, 2015). Liu et al. discovered that in human cervical cancer cells, the expression of Bcl-2 increased, while the expression of the pro-apoptotic protein bax decreased (Liu et al., 2013). Trop-2 inhibits apoptosis by directly upregulating Bcl-2 expression and suppressing bax activation (Liu et al., 2013; Chen et al., 2012). Trop-2 stimulates the expression of cell cycle regulators such as cyclin D1, cyclin E, CDK2, and CDK4 by modulating the ERK1/2 pathway, thereby promoting the G1-S and G2-M transitions, accelerating cell cycle progression, and ultimately enhancing cell proliferation (Liu et al., 2013; Cubas et al., 2010).

Trop-2 inhibits cell adhesion to fibronectin by increasing the binding of β1 integrin to the adapter molecule receptor for activated C kinase one and activating the activity of tyrosine kinase c-Src and focal adhesion kinase, leading to increased invasiveness of cancer cells (Trerotola et al., 2012). Overexpressed Trop-2 forms a complex with insulin-like growth factor 1, which inhibits the insulin-like growth factor 1 receptor signaling pathway. This inhibition suppresses the activation of β-catenin/Slug gene expression, thereby mediating the proliferation and migration of tumor cells (Pavšič et al., 2015). Trop-2 also can restrict the expression of the calcium-dependent cell adhesion molecule E-cadherin, and induces the loss of intercellular adhesion (Liu et al., 2013; Tang et al., 2011). Consequently, these effects promote the epithelial-mesenchymal transition (EMT). In solid tumors, the transition of epithelial cells to mesenchymal cells is a crucial early step in tumor invasion and metastasis (Slabáková et al., 2011). Trop-2 has emerged as a promising new molecular target for ADC due to its differential expression in normal versus tumor tissues and its internalization activity.

### 4.1 ADCs targeting Trop-2

Sacituzumab govitecan (SG, IMMU-132, Immunomedics, hRS7-SN38, Trodelvy^®^) is the first FDA approved anti-Trop-2 ADC that consists of hRS7, conjugated with SN-38 (a topoisomerase I inhibitor derived from irinotecan) through the cleavable CL2A linker (Starodub et al., 2015). Compared to the concentration of SN-38 released by irinotecan, SG can deliver a higher concentration of SN-38 to target tumor cells and release it extracellularly within the tumor microenvironment, providing a bystander effect that maximizes therapeutic efficacy (Starodub et al., 2015; Goldenberg et al., 2014). SG has been approved for the treatment of metastatic triple-negative breast cancer (TNBC), hormone receptor (HR)+/HER2- BC and metastatic UC. The feasibility of SG for treating r/mCC has also garnered significant attention. Research by Zeybek et al. has demonstrated that Trop-2-positive cervical cancer cell lines and xenograft models exhibit high sensitivity to SG (Zeybek et al., 2020).

EVER-132-003 (NCT05119907) is a multicenter, single-arm, open-label phase II study evaluating SG in patients with solid tumors (An et al., 2024). Cohort C of this study enrolled 18 Chinese adult patients with r/mCC who had disease progression after receiving ≥1L of systemic therapy. Patients were administered 10 mg/kg SG as a monotherapy intravenously on Day 1 and Day 8 of a 21-day cycle. Interim analysis data demonstrated encouraging antitumor activity of SG in r/mCC patients with an ORR of 50% and a median DoR of 9.2 months. The DCR was 94% and the median PFS in this cohort was 8.1 months. Similar efficacy was also observed in patients who had previously received immunotherapy. The most common TRAEs were decrease in neutrophil and WBC count, and anemia. SG may be extensively explored for its safety and efficacy in the cervical cancer population. Lee et al. have found that SG can increase the activity of forkhead box O3a, which may interact with Trop-2. This could potentially inhibit PD-L1 expression in breast tumors by activating natural killer cells (Lee et al., 2023). This suggests that SG may enhance the efficacy of immune checkpoint inhibitors (ICIs), making the combination therapy of Trop-2 ADCs and ICIs a recent research focus.

Sacituzumab tirumotecan, also known as sac-TMT, SKB-264, or MK-2870, is a novel anti-Trop2 ADC that was developed using 2-methylsulfonyl pyrimidine as the linker to conjugate its payload (KL610023), which is a topoisomerase I inhibitor belotecan derivative with a bystander effect, and can arrest cell cycle at the G2/S stage after its internalization, leading to cell death (Cheng et al., 2022; Lee et al., 1998). Compared to SG, SKB-264 exhibits a longer half-life, enhanced targeting efficacy, and superior anti-tumor activity. At the same dosage, the exposure of SKB-264 in tumor tissues is 4.6 times greater than that of SG (Cheng et al., 2022). SKB-264 is currently approved in China for the treatment of locally advanced or meta3ic TNBC in adults who have received >2L of systemic therapy and EGFR mutation-positive locally advanced or metastatic non-squamous NSCLC in adults who progressed after EGFR tyrosine kinase inhibitor therapy and platinum-based chemotherapy.

An ongoing phase II basket study (NCT05642780) is evaluating the efficacy and safety of SKB-264 in combination with pembrolizumab for the treatment of r/mCC. The study enrolled patients with r/mCC who had progressed during or after platinum-based doublet chemotherapy and had received no more than 2 L of systemic therapy for r/m disease. The ORR was 57.9%, the median DoR has not yet been reached, and the 6-month DoR rate was 82.1%. Responses has still been observed in patients who have previously undergone anti-PD-1 therapy (ORR was 68.8%) (Wang et al., 2024). The most common grade ≥3 TRAEs were neutrophil count decreased, anemia and WBC decreased. SKB-264 in combination with pembrolizumab has demonstrated promising and durable anti-tumor activity in cervical cancer, with a manageable safety profile. A phase III randomized, open-label, multicenter study (NCT06459180) evaluated the efficacy and safety of SKB-264 monotherapy versus treatment of physician’s choice as 2L therapy in patients with r/mCC. The study is ongoing, and no preliminary data have been disclosed.

## 5 Mesothelin

Mesothelin is a differentiation antigen expressed on normal mesothelial cells and is overexpressed in several human malignancies, including mesothelioma, ovarian adenocarcinoma, and pancreatic adenocarcinoma (Hassan et al., 2004). Mesothelin may be involved in tumor initiation, progression, invasion, and metastasis through multiple signaling pathways. Mesothelin binds to mucin16/carbohydrate antigen 125 (Kaneko et al., 2009), downregulates Dickkopf-1 (an inhibitor of the Wnt signaling pathway) through the serum and glucocorticoid-regulated kinase 3 (SGK3)/forkhead box O3 (FoxO3) signaling pathway, and activates the Wnt/β-catenin axis, thereby promoting cancer cell metastasis (Huo et al., 2021). The overexpression of mesothelin activates the PI3K/AKT, ERK1/2, and c-Jun N-terminal kinase (JNK) signaling pathways (Wang et al., 2012). The downstream effects of AKT and ERK1/2 signaling include the inhibition of pro-apoptotic proteins such as Bim, Bad, and Bax, as well as the stimulation of anti-apoptotic proteins like Bcl-xl and Bcl-2, thereby suppressing cellular apoptosis (Tang et al., 2013). The PI3K/AKT, ERK1/2, and JNK pathways can also enhance the expression of matrix metalloprotease 7, thereby promoting cell migration and invasion (Chang et al., 2012). The matrix metalloprotease 7 pathway can be triggered through the SGK3/FoxO3 and p38 pathways as well (Chen et al., 2013). Additionally, the overexpression of mesothelin leads to the activation of the p38, NF-κB and signal transducers and activators of transcripition 3 (STAT3) signaling pathways. The downstream effects of NF-κB include increasing the production of IL-6 and enhancing tumor cell proliferation and survival through auto/paracrine IL-6/sIL-6R trans-signaling (Bharadwaj et al., 2011). Constitutive activation of STAT3 results in increased expression of cyclin E and the formation of the cyclin E/CDK2 complex, promoting the G1-S transition (Faust et al., 2022).

Researches has found that mesothelin is highly expressed in cervical cancer patients, particularly in those with non-squamous cell carcinoma (Jöhrens et al., 2019; He et al., 2019; Takamizawa et al., 2022). High mesothelin expression was associated with poor OS in patients with common histological cervical cancer types (Takamizawa et al., 2022). Given the limited expression of mesothelin in normal tissues, targeting it for the treatment of cervical cancer is a viable strategy. Although clinical trials of mesothelin-targeting agents have primarily focused on pleural mesothelioma, ovarian cancer, and pancreatic cancer, preliminary efficacy has been observed with certain mesothelin-targeting ADCs in cervical cancer patients. This suggests mesothelin-targeting ADCs as promising additional therapeutic strategies for cervical cancer.

### 5.1 ADCs targeting mesothelin

RC88 is a novel ADC comprising the humanized anti-mesothelin antibody via a cleavable linker to the cytotoxic agent MMAE. A single-arm, open-label, multicenter phase I/II study (NCT04175847) evaluated the safety and efficacy of RC88 in patients with mesothelin-expressing advanced solid tumor (Liu et al., 2024). As of 19 December 2023, 164 patients with mesothelin-expressing advanced malignant solid tumors that have failed after standard therapies were enrolled, including 18 cervical cancer patients progressed on previous systemic therapy. In 17 patients with one post-baseline tumor assessment, 11 (64.7%) had received ≥ 2L of prior therapies, 12 (70.5%) had prior platinum-doublet chemotherapy and PD-(L)1 inhibitor. In cervical cancer cohort, the ORR was 35.3%. The most frequent TRAEs were WBC count decreased, neutrophil count decreased, anemia, nausea, and aspartate aminotransferase increased.

Anetumab ravtansine (AR, BAY94-9343) is a mesothelin-targeting ADC that consists of a human anti-mesothelin antibody (MF-T), a disulfide-containing linker and a maytansinoid tubulin inhibitor DM4 (Golfier et al., 2014). Although there are currently no clinical trial data demonstrating the efficacy of AR in treating cervical cancer, AR showed a substantial dose-dependent therapeutic efficiency in a xenotransplant model for cervical cancer in SCID mice (hela cell tumors) (Jöhrens et al., 2019). Applying AR at a dose of 10 mg/kg twice weekly induced complete tumor regression in 88% of animals within 6 weeks. A first-in-human, multicenter phase I dose-escalation and expansion study (NCT01439152) of AR in patients with advanced or metastatic solid tumors revealed that the most common drug-related adverse events were fatigue, nausea, diarrhea, anorexia, vomiting, peripheral sensory neuropathy, and keratitis/keratopathy (Hassan et al., 2020). It warrants further clinical investigation to evaluate the therapeutic value and long-term benefits of AR in patients with r/mCC.

## 6 Nectin cell adhesion molecule 4 (Nectin-4)

Nectin-4 is a Ca^2+^-independent, type I transmembrane, immunoglobulin-like cell adhesion molecule that exhibits restricted physiological expression primarily in embryonic and placental tissues, but is frequently overexpressed in multiple human malignancies (Rikitake et al., 2012). It is involved in various molecular pathways related to tumor cell adhesion, proliferation, migration, and angiogenesis, making it a novel biomarker and therapeutic target for cancer. Nectin-4 exists in both soluble and membrane-bound forms. Under hypoxic conditions, a disintegrin and metalloproteinase can cleave the extracellular domain of this membrane protein from the cell surface, releasing soluble Nectin-4 (Buchanan et al., 2017).

Nectin-4 participates in several critical processes in tumors through the key signaling pathway of PI3K/AKT. The interaction between soluble Nectin-4 and integrin β4 on endothelial cells can regulate the transcriptional activity of Src, PI3K, AKT, and endothelial NO synthase, inducing the formation of NO mediated by the PI3K/AKT signaling pathway, thereby promoting tumor angiogenesis (Zhang et al., 2019; Siddharth et al., 2018). In BC, Nectin-4 and HER2 engage in cis-interactions, activating the PI3K/AKT signaling pathway to enhance DNA synthesis. Soluble Nectin-4 further promotes inositol polyphosphate 4-phosphatase type II-dependent lysosomal degradation via the PI3K/AKT axis, which activates the Wnt/β-catenin signaling pathway, driving the proliferation and metastasis of BC cells (Kedashiro et al., 2019; Siddharth et al., 2017; Rodgers et al., 2023). Additionally, in osteosarcoma, Nectin-4 directly downregulates the microRNA miR-520c-3p, activating the PI3K/AKT/NF-κB pathway and promoting tumor progression and metastasis (Liu et al., 2022). The upregulation of Nectin-4 can also activate the Rac1 (Ras-related C3 botulinum toxin substrate 1) signaling pathway through the PI3K/AKT axis. Activated Rac1 stimulates p21-activated kinases and JNK, which initiate cytoskeletal reorganization and regulate cell adhesion, migration, and proliferation (Zhang et al., 2016).

In addition to the PI3K/AKT pathway, Nectin-4 is also involved in the transduction of other signaling pathways. The extracellular domain of Nectin-4 also interacts in cis with the prolactin receptor, which is essential for mammary follicle development, activating the JAK2–STAT5a signaling pathway, thereby regulating the growth of tumor cells (Maruoka et al., 2017; Tan and Nevalainen, 2008). Nectin-4 regulates intercellular adhesion, remodels the actin cytoskeleton, triggers EMT, enhances the driving force for tumor cell pseudopodia extension, and ultimately leads to tumor development and metastasis (Samanta and Almo, 2015).

Nectin-4’s involvement in cervical cancer pathogenesis is poorly characterized, with sparse data on its expression dynamicsHalle et al. performed IHC analysis of Nectin-4 membrane expression in tumor specimens from 525 cervical cancer patients, identifying high expression levels in 4% of cases (Halle et al., 2024). In the cervical cancer expansion cohort of a clinical trial for a novel Nectin-4-targeting ADC, the detection rate of Nectin-4 expression was 89.67%, and the detection rate of Nectin-4 tumor cell staining intensity of 3+ was 67.82% (Zhai et al., 2024). Emerging evidence suggests that Nectin-4 may activate double-strand DNA repair pathways in cervical cancer stem cells, thereby promoting malignant progression. Supporting this mechanism, Nayak et al. demonstrated that quinacrine nanoparticles-functioning as Nectin-4 inhibitors-effectively suppress both cellular proliferation and DNA damage response in cervical cancer stem cells (Nayak et al., 2019). Nectin-4 could serve as an effective target for cervical cancer, and Nectin-4-targeting ADCs have already demonstrated promising efficacy in cervical cancer patients.

### 6.1 ADCs targeting Nectin-4

9MW2821 is novel anti-Nectin-4 ADC independently developed by Mavis Biologics, featuring a site specifically conjugated humanized antibod, an enzymatically cleavable valine–citrulline linker and MMAE as the payload (Fang et al., 2024). A first-in-human, open label, multicenter phase I/IIa study (NCT05216965) evaluating the safety and preliminary efficacy of 9MW2821 enrolled 274 patients with Nectin-4-positive solid tumors who failed ≥1L of systemic therapy (Zhang et al., 2025). In 53 evaluable r/mCC patients treated with 9MW2821 at a dose of 1.25 mg/kg, the ORR was 32.1%, and the DCR reached 81.1%. The median DoR, PFS, and OS were 6.3 months, 3.9 months, and 16.0 months, respectively. For patients treated with ICIs previously, comparable clinical benefit was observed. In the exploratory analysis, 69.8% of patients exhibited moderate-to-high Nectin-4 expression based on H-SCORE evaluation. 9MW2821 is the first Nectin-4-targeted ADC to demonstrate antitumor activity in patients with cervical cancer. In the 1.25 mg/kg dose group, the most common grade ≥3 TEAEs were neutrophil count decreased, WBC count decreased, anemia, gammaglutamyl transferase increased rash and peripheral sensory neuropathy (Zhang et al., 2025).

ADRX-0706 is a next-generation Nectin-4-targeting ADC with a drug-to-antibody ratio of 8 and enhanced bystander effect. It consists of a fully human IgG1κ antibody conjugated to a proprietary tubulin inhibitor (AP052) via a cleavable linker using Adcentrx’s i-Conjugation™ technology (Hau et al., 2024). ADRX-0706 demonstrates high selectivity and potent antitumor activity across a variety of tumor models. In a mouse clinical trial of patient-derived cervical cancers with a range of Nectin-4 expression levels, ADRX-0706 reaches a 73% ORR (98). ADRX-0706 is currently in phase Ia/b clinical study (NCT06036121), primarily evaluating its safety and efficacy in solid tumors including cervical cancer, UC, and TNBC. No preliminary data has been disclosed yet, but its therapeutic potential in the cervical cancer population is promising.

## 7 Conclusion

In summary, this article reviews potential targets for ADCs in cervical cancer and elucidates their mechanisms of action in tumor biology. By summarizing the latest clinical trial results of representative ADCs in cervical cancer, we highlight their potential to transform the treatment landscape for patients with recurrent or metastatic disease. Several ADCs, including TV, T-DXd, have already demonstrated significant therapeutic activity in patients with advanced cervical cancer.

While this review comprehensively analyzed the clinical efficacy of ADCs, it is important to note that detailed pharmacokinetic (e.g., clearance rates, volume of distribution) and pharmacodynamic (e.g., target engagement biomarkers) data from the included trials were not publicly available. This gap limits our ability to fully correlate drug exposure with therapeutic outcomes or adverse events. Future trials should prioritize reporting these parameters to facilitate mechanistic understanding of ADCs in cervical cancer.

Future research should focus on identifying novel TAAs and optimizing ADC design through payload diversification and antibody engineering to enhance both efficacy and safety. It is imperative to discover broad-spectrum biomarkers based on ADC mechanisms or pharmacodynamic effects, enabling biomarker-guided patient stratification for precision medicine. Additionally, exploring ADC-based combination therapies may further improve clinical outcomes while mitigating drug resistance. A comprehensive understanding of the biological roles of TAAs in tumor angiogenesis, host immune responses, and multiple cellular signaling pathways will provide critical insights for developing combination therapeutic strategies. Given Trop-2’s regulatory role in cervical cancer progression and host immune responses, clinical trials have been initiated to evaluate the efficacy and safety of combining Trop-2-directed ADCs with ICIs. The synergistic effect of mesothelin and mucin16 in malignant peritoneal metastasis suggests that developing bispecific ADCs may represent a promising strategy to enhance therapeutic efficacy. Furthermore, future research should explore combination therapies pairing ADCs with molecularly targeted agents to simultaneously inhibit multiple signaling pathways. For instance, combining Trop-2-directed ADCs with ERK phosphorylation inhibitors or Nectin-4-targeting ADCs with AKT inhibitors may yield more effective antitumor treatment strategies.

ADCs retain tremendous untapped potential in cervical cancer treatment. As this field continues to advance, ADCs are poised to play an increasingly pivotal role in therapeutic strategies, offering renewed hope for improving survival rates and clinical outcomes for patients worldwide.

## References

[B1] Abu-Rustum N. R. Yashar C. M. Arend R. Barber E. Bradley K. Brooks R. (2023). NCCN Guidelines® insights: cervical cancer, version 1.2024. J. Natl. Compr. Canc. Netw. 21 (12), 1224–1233. 10.6004/jnccn.2023.0062 38081139

[B2] Ahmadi S. E. Shabannezhad A. Kahrizi A. Akbar A. Safdari S. M. Hoseinnezhad T. (2023). Tissue factor (coagulation factor III): a potential double-edge molecule to be targeted and re-targeted toward cancer. Biomark. Res. 11 (1), 60. 10.1186/s40364-023-00504-6 37280670 PMC10242999

[B3] An J. Li G. Zhang Y. Kong W. Feng M. Xu C. (2024). Sacituzumab govitecan for Chinese patients with recurrent/metastatic cervical cancer: interim analysis of the phase II basket study EVER-132-003. Gynecol. Oncol. 190, S22. 10.1016/j.ygyno.2024.07.038 40972504

[B4] Arkhipov A. Shan Y. Kim E. T. Dror R. O. Shaw DEJE (2013). Her2 activation mechanism reflects evolutionary preservation of asymmetric ectodomain dimers in the human EGFR family. Elife 2, e00708. 10.7554/eLife.00708 23878723 PMC3713454

[B5] Bharadwaj U. Marin-Muller C. Li M. Chen C. Yao Q. J. (2011). Mesothelin overexpression promotes autocrine IL-6/sIL-6R trans-signaling to stimulate pancreatic cancer cell proliferation. Carcinogenesis 32 (7), 1013–1024. 10.1093/carcin/bgr075 21515913 PMC3128561

[B6] Boussios S. Seraj E. Zarkavelis G. Petrakis D. Kollas A. Kafantari A. (2016). Management of patients with recurrent/advanced cervical cancer beyond first line platinum regimens: where do we stand? A lit. Rev. 108, 164–174. 10.1016/j.critrevonc.2016.11.006 27931835

[B7] Bray F. Laversanne M. Sung H. Ferlay J. Siegel R. L. Soerjomataram I. (2024). Global cancer statistics 2022: GLOBOCAN estimates of incidence and mortality worldwide for 36 cancers in 185 countries. Ca. Cancer J. Clin. 74 (3), 229–263. 10.3322/caac.21834 38572751

[B8] Breij E. C. W. de Goeij BECG Verploegen S. Schuurhuis D. H. Amirkhosravi A. Francis J. (2014). An antibody–drug conjugate that targets tissue factor exhibits potent therapeutic activity against a broad range of solid tumors. Cancer Res. 74 (4), 1214–1226. 10.1158/0008-5472.CAN-13-2440 24371232

[B9] Buchanan P. C. Boylan K. L. Walcheck B. Heinze R. Geller M. A. Argenta P. A. (2017). Ectodomain shedding of the cell adhesion molecule Nectin-4 in ovarian cancer is mediated by ADAM10 and ADAM17. J. Biol. Chem. 292 (15), 6339–6351. 10.1074/jbc.M116.746859 28232483 PMC5391762

[B10] Chang M.-C. Chen C.-A. Chen P.-J. Chiang Y.-C. Chen Y.-L. Mao T.-L. (2012). Mesothelin enhances invasion of ovarian cancer by inducing MMP-7 through MAPK/ERK and JNK pathways. Biochem. J. 442 (2), 293–302. 10.1042/BJ20110282 21999204

[B11] Chau C. H. Steeg P. S. Figg W. (2019). Antibody–drug conjugates for cancer. Lancet 394 (10200), 793–804. 10.1016/S0140-6736(19)31774-X 31478503

[B12] Chen C.-R. Xia Y.-H. Yao S.-Y. Zhang Q. Wang Y. Ji Z. (2012). Virosecurinine induces apoptosis by affecting Bcl-2 and Bax expression in human colon cancer SW480 cells. Pharmazie 67 (4), 351–354. 10.1691/ph.2012.1634 22570942

[B13] Chen S.-H. Hung W.-C. Wang P. Paul C. Konstantopoulos K. J. (2013). Mesothelin binding to CA125/MUC16 promotes pancreatic cancer cell motility and invasion via MMP-7 activation. Sci. Rep. 3 (1), 1870. 10.1038/srep01870 23694968 PMC3660778

[B14] Cheng Y. Yuan X. Tian Q. Huang X. Chen Y. Pu Y. (2022). Preclinical profiles of SKB264, a novel anti-TROP2 antibody conjugated to topoisomerase inhibitor, demonstrated promising antitumor efficacy compared to IMMU-132. Front. Oncol. 12, 951589. 10.3389/fonc.2022.951589 36620535 PMC9817100

[B15] Chiba Y. Kojima Y. Yazaki S. Yoshida H. Takamizawa S. Kitadai R. (2024). Trop-2 expression and the tumor immune microenvironment in cervical cancer. Gynecol. Oncol. 187, 51–57. 10.1016/j.ygyno.2024.04.022 38723340

[B16] Citri A. Yarden Y. J. (2006). EGF–ERBB signalling: towards the systems level. Nat. Rev. Mol. Cell Biol. 7 (7), 505–516. 10.1038/nrm1962 16829981

[B17] Coleman R. L. Lorusso D. Gennigens C. González-Martín A. Randall L. Cibula D. (2021). Efficacy and safety of tisotumab vedotin in previously treated recurrent or metastatic cervical cancer (innovaTV 204/GOG-3023/ENGOT-cx6): a multicentre, open-label, single-arm, phase 2 study. Lancet Oncol. 22 (5), 609–619. 10.1016/S1470-2045(21)00056-5 33845034

[B18] Colombo N. Dubot C. Lorusso D. Caceres M. V. Hasegawa K. Shapira-Frommer R. (2021). Pembrolizumab for persistent, recurrent, or metastatic cervical cancer. N. Engl. J. Med. 385 (20), 1856–1867. 10.1056/NEJMoa2112435 34534429

[B19] Cubas R. Zhang S. Li M. Chen C. Yao Q. J. (2010). Trop2 expression contributes to tumor pathogenesis by activating the ERK MAPK pathway. Mol. Cancer 9, 253–13. 10.1186/1476-4598-9-253 20858281 PMC2946292

[B20] Eble J. A. Niland S. (2019). The extracellular matrix in tumor progression and metastasis. Clin. Exp. Metastasis 36 (3), 171–198. 10.1007/s10585-019-09966-1 30972526

[B21] Fang J. Gu L. Zhu N. Tang H. Alvarado C. S. Zhou M. J. B. (2008). Tissue factor/FVIIa activates Bcl-2 and prevents doxorubicin-induced apoptosis in neuroblastoma cells. BMC Cancer 8, 69–11. 10.1186/1471-2407-8-69 18325115 PMC2275284

[B22] Fang P. You M. Cao Y. Feng Q. Shi L. Wang J. (2024). Development and validation of bioanalytical assays for the quantification of 9MW2821, a nectin-4-targeting antibody–drug conjugate. J. Pharm. Biomed. Anal. 248, 116318. 10.1016/j.jpba.2024.116318 38908237

[B23] Faust J. R. Hamill D. Kolb E. A. Gopalakrishnapillai A. Barwe S. P. J. (2022). Mesothelin: an immunotherapeutic target beyond solid tumors. Cancers (Basel). 14 (6), 1550. 10.3390/cancers14061550 35326701 PMC8946840

[B24] Giudice E. Camarda F. Salutari V. Ricci C. Nero C. Carbone M. V. (2021). Tisotumab vedotin in cervical cancer: current status and future perspectives. Oncol. Haematol. 17, 68. 10.17925/ohr.2021.17.2.68

[B25] Goldenberg D. M. Rossi E. A. Govindan S. V. Cardillo T. M. McBride W. J. Zalath M. (2014). Characterization of an anti-Trop-2-SN-38 antibody-drug conjugate (IMMU-132) with potent activity against solid cancers. Am. Soc. Clin. Oncol. 32, 3107. 10.1200/jco.2014.32.15_suppl.3107

[B26] Goldenberg D. M. Stein R. Sharkey R. M. (2018). The emergence of trophoblast cell-surface antigen 2 (TROP-2) as a novel cancer target. Oncotarget 9 (48), 28989–29006. 10.18632/oncotarget.25615 29989029 PMC6034748

[B27] Golfier S. Kopitz C. Kahnert A. Heisler I. Schatz C. A. Stelte-Ludwig B. (2014). Anetumab ravtansine: a novel mesothelin-targeting antibody–drug conjugate cures tumors with heterogeneous target expression favored by bystander effect. Mol. Cancer Ther. 13 (6), 1537–1548. 10.1158/1535-7163.MCT-13-0926 24714131

[B28] Gutierrez C. Schiff R. J. (2011). HER2: biology, detection, and clinical implications. Arch. Pathol. Lab. Med. 135 (1), 55–62. 10.5858/2010-0454-RAR.1 21204711 PMC3242418

[B29] Halle M. Ulvang M. Berg H. Woie K. Haldorsen I. Bertelsen B. (2024). 24MO TROP-2, TF and NECTIN4 as targets for ADC treatment in cervical cancer. ESMO Open 9, 103524. 10.1016/j.esmoop.2024.103524

[B30] Hassan R. Bera T. Pastan I. J. (2004). Mesothelin: a new target for immunotherapy. Clin. Cancer Res. 10 (12), 3937–3942. 10.1158/1078-0432.CCR-03-0801 15217923

[B31] Hassan R. Blumenschein G. R. Jr Moore K. N. Santin A. D. Kindler H. L. Nemunaitis J. J. (2020). First-in-human, multicenter, phase I dose-escalation and expansion study of anti-mesothelin antibody–drug conjugate anetumab ravtansine in advanced or metastatic solid tumors. J. Clin. Oncol. 38 (16), 1824–1835. 10.1200/JCO.19.02085 32213105 PMC7255978

[B32] Hau A. M. Shahmoradgoli M. Lee D. J. Sisson W. Wang A. Challita P. P. (2024). Abstract 1891: preclinical characterization of ADRX-0706: a next-generation anti-Nectin-4 antibody-drug conjugate with improved therapeutic window. Cancer Res. 84 (6_Suppl), 1891. 10.1158/1538-7445.am2024-1891

[B33] He Y. Zhao H. Li X.-M. Yin C.-H. Wu Y.-M. J. G. (2019). Use of mesothelin as a tumor-associated antigen in cervical squamous cell carcinoma. Gene 690, 30–37. 10.1016/j.gene.2018.12.029 30583024

[B34] Heitz N. Greer S. C. Halford Z. (2023). A review of tisotumab vedotin-tftv in recurrent or metastatic cervical cancer. Ann. Pharmacother. 57 (5), 585–596. 10.1177/10600280221118370 35962528

[B35] Hisada Y. Mackman N. (2019). Tissue factor and cancer: regulation, tumor growth, and metastasis. Seminars thrombosis hemostasis 45, 385–395. 10.1055/s-0039-1687894 PMC6546519 31096306

[B36] Hong D. S. Concin N. Vergote I. de Bono J. S. Slomovitz B. M. Drew Y. (2020). Tisotumab vedotin in previously treated recurrent or metastatic cervical cancer. Clin. Cancer Res. 26 (6), 1220–1228. 10.1158/1078-0432.CCR-19-2962 31796521

[B37] Huo Q. Xu C. Shao Y. Yu Q. Huang L. Liu Y. (2021). Free CA125 promotes ovarian cancer cell migration and tumor metastasis by binding Mesothelin to reduce DKK1 expression and activate the SGK3/FOXO3 pathway. Int. J. Biol. Sci. 17 (2), 574–588. 10.7150/ijbs.52097 33613114 PMC7893585

[B38] Itkin B. Garcia A. Straminsky S. Adelchanow E. D. Pereyra M. Haab G. A. (2021). Prevalence of HER2 overexpression and amplification in cervical cancer: a systematic review and meta-analysis. PLoS One 16 (9), e0257976. 10.1371/journal.pone.0257976 34591928 PMC8483403

[B39] Jöhrens K. Lazzerini L. Barinoff J. Sehouli J. Cichon G. J. A. (2019). Mesothelin as a target for cervical cancer therapy. Arch. Gynecol. Obstet. 299, 211–216. 10.1007/s00404-018-4933-z 30324544

[B40] Kaneko O. Gong L. Zhang J. Hansen J. K. Hassan R. Lee B. (2009). A binding domain on mesothelin for CA125/MUC16. J. Biol. Chem. 284 (6), 3739–3749. 10.1074/jbc.M806776200 19075018 PMC2635045

[B41] Kantak S. Faggioni R. Cai A. G. Bhatti M. M. Li J. Vainshtein I. (2024). Preclinical characterization of XB002, an anti–tissue factor antibody–drug conjugate for the treatment of solid tumors. Mol. Cancer Ther. 24, 251–260. 10.1158/1535-7163.mct-24-0002 PMC11791478 39494690

[B42] Kasthuri R. S. Taubman M. B. Mackman N. J. (2009). Role of tissue factor in cancer. J. Clin. Oncol. 27 (29), 4834–4838. 10.1200/JCO.2009.22.6324 19738116 PMC2764391

[B43] Kedashiro S. Sugiura A. Mizutani K. Takai Y. J. (2019). Nectin-4 cis-interacts with ErbB2 and its trastuzumab-resistant splice variants, enhancing their activation and DNA synthesis. DNA Synth. 9 (1), 18997. 10.1038/s41598-019-55460-9 PMC6908695 31831814

[B44] Kocatürk B. Versteeg H. H. (2013). Tissue factor‐integrin interactions in cancer and thrombosis: every J ack has his J ill. J. Thromb. Haemost. 11, 285–293. 10.1111/jth.12222 23809132

[B45] Lee J.-H. Lee J.-M. Kim J.-K. Ahn S.-K. Lee S.-J. Kim M.-Y. (1998). Antitumor activity of 7-[2-(N-isopropylamino) ethyl]-(20 S)-camptothecin, CKD602, as a potent DNA topoisomerase I inhibitor. Arch. Pharm. Res. 21, 581–590. 10.1007/BF02975379 9875499

[B46] Lee Y. M. Chen Y. H. Ou D. L. Hsu C. L. Liu J. H. Ko J. Y. (2023). SN‐38, an active metabolite of irinotecan, enhances anti‐PD‐1 treatment efficacy in head and neck squamous cell carcinoma. J. Pathol. 259 (4), 428–440. 10.1002/path.6055 36641765

[B47] Leppert U. Eisenreich A. (2015). The role of tissue factor isoforms in cancer biology. Int. J. Cancer 137 (3), 497–503. 10.1002/ijc.28959 24806794

[B48] Li B. Meric-Bernstam F. Bardia A. Naito Y. Siena S. Aftimos P. (2023). 654O Efficacy and safety of trastuzumab deruxtecan (T-DXd) in patients (pts) with solid tumors harboring specific HER2-activating mutations (HER2m): primary results from the international phase II DESTINY-PanTumor01 (DPT-01) study. Ann. Oncol. 34, S459–S460. 10.1016/j.annonc.2023.09.1840

[B49] Lin J. C. Wu Y. Y. Wu J. Y. Lin T. C. Wu C. T. Chang Y. L. (2012). TROP2 is epigenetically inactivated and modulates IGF‐1R signalling in lung adenocarcinoma. EMBO Mol. Med. 4 (6), 472–485. 10.1002/emmm.201200222 22419550 PMC3443948

[B50] Lipinski M. Parks D. R. Rouse R. V. Herzenberg L. (1981). Human trophoblast cell-surface antigens defined by monoclonal antibodies. Proc. Natl. Acad. Sci. U. S. A. 78 (8), 5147–5150. 10.1073/pnas.78.8.5147 7029529 PMC320350

[B51] Liu T. Liu Y. Bao X. Tian J. Liu Y. Yang X. J. (2013). Overexpression of TROP2 predicts poor prognosis of patients with cervical cancer and promotes the proliferation and invasion of cervical cancer cells by regulating ERK signaling pathway. PLoS One 8 (9), e75864. 10.1371/journal.pone.0075864 24086649 PMC3785439

[B52] Liu Y. Li G. Yang R. Huang Y. Luo S. Dang Q. (2024). The efficacy and safety of RC88 in patients with ovarian cancer, non-squamous-non-small-cell lung-carcinoma and cervical cancer: results from a first-in-human phase 1/2 study. Am. Soc. Clin. Oncol. 42, 5551. 10.1200/jco.2024.42.16_suppl.5551

[B53] Liu Y. Li G. Zhang Y. Li L. Zhang Y. Huang X. (2022). Nectin-4 promotes osteosarcoma progression and metastasis through activating PI3K/AKT/NF-κB signaling by down-regulation of miR-520c-3p. Cancer Cell Int. 22 (1), 252. 10.1186/s12935-022-02669-w 35953862 PMC9367085

[B54] Mackman N. (2004). Role of tissue factor in hemostasis, thrombosis, and vascular development. Arterioscler. Thromb. Vasc. Biol. 24 (6), 1015–1022. 10.1161/01.ATV.0000130465.23430.74 15117736

[B55] Maruoka M. Kedashiro S. Ueda Y. Mizutani K. Takai Y. J. (2017). Nectin-4 co-stimulates the prolactin receptor by interacting with SOCS1 and inhibiting its activity on the JAK2-STAT5a signaling pathway. J. Biol. Chem. 292 (17), 6895–6909. 10.1074/jbc.M116.769091 28258213 PMC5409460

[B56] Meric-Bernstam F. Makker V. Oaknin A. Oh D.-Y. Banerjee S. González-Martín A. (2024). Efficacy and safety of trastuzumab deruxtecan in patients with HER2-expressing solid tumors: primary results from the DESTINY-PanTumor02 phase II trial. J. Clin. Oncol. 42 (1), 47–58. 10.1200/JCO.23.02005 37870536 PMC10730032

[B57] Nayak A. Das S. Nayak D. Sethy C. Narayan S. Kundu C. N. (2019). Nanoquinacrine sensitizes 5-FU-resistant cervical cancer stem-like cells by down-regulating Nectin-4 via ADAM-17 mediated NOTCH deregulation. Cell. Oncol. 42 (2), 157–171. 10.1007/s13402-018-0417-1 PMC12994334 30603978

[B58] Ogitani Y. Aida T. Hagihara K. Yamaguchi J. Ishii C. Harada N. (2016). DS-8201a, a novel HER2-targeting ADC with a novel DNA topoisomerase I inhibitor, demonstrates a promising antitumor efficacy with differentiation from T-DM1. Clin. Cancer Res. 22 (20), 5097–5108. 10.1158/1078-0432.CCR-15-2822 27026201

[B59] Park W. Zhang J. Dayyani F. Shan J. Liu R. Guo R. (2024). Phase I/II first-in-human study to evaluate the safety and efficacy of tissue factor-ADC MRG004A in patients with solid tumors. Am. Soc. Clin. Oncol. 42, 3002. 10.1200/jco.2024.42.16_suppl.3002

[B60] Pavšič M. Ilc G. Vidmar T. Plavec J. Lenarčič B. J. (2015). The cytosolic tail of the tumor marker protein Trop2-a structural switch triggered by phosphorylation. Sci. Rep. 5 (1), 10324. 10.1038/srep10324 25981199 PMC4434849

[B61] Platform MCPP, monoclonal Abs A-T (2025). Tissue factor (TF/CD142)–A potential new horizon for pancreatic cancer therapy?

[B62] Popescu N. C. King C. R. Kraus M. H. (1989). Localization of the human erbB-2 gene on normal and rearranged chromosomes 17 to bands q12–21.32. Genomics 4 (3), 362–366. 10.1016/0888-7543(89)90343-1 2565881

[B63] Porras G. O. R. Nogueda J. C. Chacón A. P. (2018). Chemotherapy and molecular therapy in cervical cancer. Rep. Pract. Oncol. Radiother. 23 (6), 533–539. 10.1016/j.rpor.2018.09.002 30534017 PMC6277350

[B64] Rikitake Y. Mandai K. Takai Y. J. (2012). The role of nectins in different types of cell–cell adhesion. J. Cell Sci. 125 (16), 3713–3722. 10.1242/jcs.099572 23027581

[B65] Rodgers S. J. Mitchell C. A. Ooms L. M. (2023). The mechanisms of class 1A PI3K and Wnt/β-catenin coupled signaling in breast cancer. breast cancer 51 (4), 1459–1472. 10.1042/BST20220866 PMC10586779 37471270

[B66] Rubin I. Yarden Y. (2001). The basic biology of HER2. Ann. Oncol. 12, S3–S8. 10.1093/annonc/12.suppl_1.s3 11521719

[B67] Samanta D. Almo S. C. (2015). Nectin family of cell-adhesion molecules: structural and molecular aspects of function and specificity. Cell Mol. Life Sci. 72, 645–658. 10.1007/s00018-014-1763-4 25326769 PMC11113404

[B68] Schaffner F. Ruf W. (2009). Tissue factor and PAR2 signaling in the tumor microenvironment. Arterioscler. Thromb. Vasc. Biol. 29 (12), 1999–2004. 10.1161/ATVBAHA.108.177428 19661489 PMC2806842

[B69] Shu J. Zhu T. Huang Y. Xu Q. Guo R. Liu H. (2024). 720MO IBI354 (anti-HER2 antibody-drug conjugate [ADC]) in patients (pts) with advanced gynecological cancers (Gynecol C): results from a phase I study. Ann. Oncol. 35, S551. 10.1016/j.annonc.2024.08.782

[B70] Shvartsur A. Bonavida B. J. (2015). Trop2 and its overexpression in cancers: regulation and clinical/therapeutic implications. Genes Cancer 6 (3-4), 84–105. 10.18632/genesandcancer.40 26000093 PMC4426947

[B71] Siddharth S. Goutam K. Das S. Nayak A. Nayak D. Sethy C. (2017). Nectin-4 is a breast cancer stem cell marker that induces WNT/β-catenin signaling via Pi3k/Akt axis. Int. J. Biochem. Cell Biol. 89, 85–94. 10.1016/j.biocel.2017.06.007 28600142

[B72] Siddharth S. Nayak A. Das S. Nayak D. Panda J. Wyatt M. D. (2018). The soluble nectin-4 ecto-domain promotes breast cancer induced angiogenesis via endothelial Integrin-β4. Int. J. Biochem. Cell Biol. 102, 151–160. 10.1016/j.biocel.2018.07.011 30056265

[B73] Slabáková E. Pernicová Z. Slavíčková E. Staršíchová A. Kozubík A. Souček K. J. (2011). TGF‐β1‐induced EMT of non‐transformed prostate hyperplasia cells is characterized by early induction of SNAI2/Slug. Prostate 71 (12), 1332–1343. 10.1002/pros.21350 21321977

[B74] Spector N. L. Blackwell K. L. (2009). Understanding the mechanisms behind trastuzumab therapy for human epidermal growth factor receptor 2–positive breast cancer. J. Clin. Oncol. 27 (34), 5838–5847. 10.1200/JCO.2009.22.1507 19884552

[B75] Starodub A. N. Ocean A. J. Shah M. A. Guarino M. J. Picozzi Jr V. J. Vahdat L. T. (2015). First-in-human trial of a novel anti-Trop-2 antibody-SN-38 conjugate, sacituzumab govitecan, for the treatment of diverse metastatic solid tumors. Clin. Cancer Res. 21 (17), 3870–3878. 10.1158/1078-0432.CCR-14-3321 25944802 PMC4558321

[B76] Suzuki M. Yagishita S. Sugihara K. Ogitani Y. Nishikawa T. Ohuchi M. (2021). Visualization of intratumor pharmacokinetics of [fam-] trastuzumab deruxtecan (DS-8201a) in HER2 heterogeneous model using phosphor-integrated dots imaging analysis. Clin. Cancer Res. 27 (14), 3970–3979. 10.1158/1078-0432.CCR-21-0397 33980613

[B77] Takamizawa S. Yazaki S. Kojima Y. Yoshida H. Kitadai R. Nishikawa T. (2022). High mesothelin expression is correlated with non-squamous cell histology and poor survival in cervical cancer: a retrospective study. BMC Cancer 22 (1), 1215. 10.1186/s12885-022-10277-0 36434635 PMC9701073

[B78] Tan S.-H. Nevalainen M. T. (2008). Signal transducer and activator of transcription 5A/B in prostate and breast cancers. Endocr. Relat. Cancer 15 (2), 367–390. 10.1677/ERC-08-0013 18508994 PMC6036917

[B79] Tang B. Peng Z.-H. Yu P.-W. Yu G. Qian F. J. (2011). Expression and significance of Cx43 and E-cadherin in gastric cancer and metastatic lymph nodes. Med. Oncol. 28, 502–508. 10.1007/s12032-010-9492-5 20373058

[B80] Tang Z. Qian M. Ho M. J. (2013). The role of mesothelin in tumor progression and targeted therapy. Anticancer. Agents Med. Chem. 13 (2), 276–280. 10.2174/1871520611313020014 22721387 PMC3568227

[B81] Tarantino P. Carmagnani Pestana R. Corti C. Modi S. Bardia A. Tolaney S. M. (2022). Antibody–drug conjugates: smart chemotherapy delivery across tumor histologies. Ca. Cancer J. Clin. 72 (2), 165–182. 10.3322/caac.21705 34767258

[B82] Tewari K. S. Sill M. W. Long I. I. I. H. J. Penson R. T. Huang H. Ramondetta L. M. (2014). Improved survival with bevacizumab in advanced cervical cancer. N. Engl. J. Med. 370 (8), 734–743. 10.1056/NEJMoa1309748 24552320 PMC4010094

[B83] Toumi M. Mathe I. J. (2023). Trop2: a key player in oncology—from research to clinical application. J. Genet. Eng. 5, 124–132. Available online at: https://www.researchgate.net/profile/Ru-Kuang/publication/374219515_Trop2_A_Key_Player_in_Oncology_-From_Research_to_Clinical_Application/links/651424b437d0df2448f11436/Trop2-A-Key-Player-in-Oncology-From-Research-to-Clinical-Application.pdf.

[B84] Trerotola M. Li J. Alberti S. Languino L. R. (2012). Trop‐2 inhibits prostate cancer cell adhesion to fibronectin through the β1 integrin‐RACK1 axis. J. Cell. Physiol. 227 (11), 3670–3677. 10.1002/jcp.24074 22378065 PMC3369113

[B85] Varughese J. Cocco E. Bellone S. Ratner E. Silasi D.-A. Azodi M. (2011). Cervical carcinomas overexpress human trophoblast cell-surface marker (Trop-2) and are highly sensitive to immunotherapy with hRS7, a humanized monoclonal anti-Trop-2 antibody. Am. J. Obstet. Gynecol. 205 (6), 567.e1–567.e5677. 10.1016/j.ajog.2011.06.093 PMC3224189 21889762

[B86] Vergote I. Martin A. G. Fujiwara K. Kalbacher E. Bagameri A. Ghamande S. (2023b). LBA9 innovaTV 301/ENGOT-cx12/GOG-3057: a global, randomized, open-label, phase III study of tisotumab vedotin vs investigator’s choice of chemotherapy in 2L or 3L recurrent or metastatic cervical cancer. Ann. Oncol. 34, S1276–S1277. 10.1016/j.annonc.2023.10.029

[B87] Vergote I. Van Nieuwenhuysen E. O'Cearbhaill R. E. Westermann A. Lorusso D. Ghamande S. (2023a). Tisotumab vedotin in combination with carboplatin, pembrolizumab, or bevacizumab in recurrent or metastatic cervical cancer: results from the innovaTV 205/GOG-3024/ENGOT-cx8 study. J. Clin. Oncol. 41 (36), 5536–5549. 10.1200/JCO.23.00720 37651655 PMC10730069

[B88] Wang J. An R. Huang Y. Zhang J. Goh J. Jiang K. (2024). 716MO Efficacy and safety of sacituzumab tirumotecan (sac-TMT) plus pembrolizumab in patients with recurrent or metastatic cervical cancer. Ann. Oncol. 35, S548–S549. 10.1016/j.annonc.2024.08.778

[B89] Wang K. Bodempudi V. Liu Z. Borrego-Diaz E. Yamoutpoor F. Meyer A. (2012). Inhibition of mesothelin as a novel strategy for targeting cancer cells. PLoS One 7 (4), e33214. 10.1371/journal.pone.0033214 22485139 PMC3317639

[B90] Xu Y. Wang Y. Gong J. Zhang X. Peng Z. Sheng X. (2021). Phase I study of the recombinant humanized anti-HER2 monoclonal antibody–MMAE conjugate RC48-ADC in patients with HER2-positive advanced solid tumors. Gastric Cancer 24 (4), 913–925. 10.1007/s10120-021-01168-7 33945049 PMC8205919

[B91] Yuan G. Li G. Li Q. Zhang Y. An R. Miao J. (2024). Evaluation of the effectiveness and safety of disitamab vedotin in HER2-expressing 2L recurrent or metastatic cervical cancer (r/mCC): interim results of RC48-C018. Am. Soc. Clin. Oncol. 42, 5528. 10.1200/jco.2024.42.16_suppl.5528

[B92] Zeybek B. Manzano A. Bianchi A. Bonazzoli E. Bellone S. Buza N. (2020). Cervical carcinomas that overexpress human trophoblast cell-surface marker (Trop-2) are highly sensitive to the antibody-drug conjugate sacituzumab govitecan. Sci. Rep. 10 (1), 973. 10.1038/s41598-020-58009-3 31969666 PMC6976591

[B93] Zhai C. Cui Y. Guo L. Chen C. Song Y. Zhong J. (2024). Progress in the study of antibody-drug conjugates for the treatment of cervical cancer. Front. Oncol. 14, 1395784. 10.3389/fonc.2024.1395784 38903711 PMC11187480

[B94] Zhang J. Liu K. Peng P. Li S. Ye Z. Su Y. (2019). Upregulation of nectin-4 is associated with ITGB1 and vasculogenic mimicry and may serve as a predictor of poor prognosis in colorectal cancer. Oncol. Lett. 18 (2), 1163–1170. 10.3892/ol.2019.10417 31423176 PMC6607174

[B95] Zhang J. Liu R. Wang S. Feng Z. Yang H. Gao S. (2025). Bulumtatug Fuvedotin (BFv, 9MW2821), a next-generation Nectin-4 targeting antibody-drug conjugate, in patients with advanced solid tumors: a first-in-human, open label, multicenter, phase Ⅰ/Ⅱ study. Ann. Oncol. 34, S464. 10.1016/j.annonc.2023.09.1845 40288679

[B96] Zhang Y. Liu S. Wang L. Wu Y. Hao J. Wang Z. (2016). A novel PI3K/AKT signaling axis mediates Nectin-4-induced gallbladder cancer cell proliferation, metastasis and tumor growth. Cancer Lett. 375 (1), 179–189. 10.1016/j.canlet.2016.02.049 26949052

[B97] Zhao X. Cheng C. Gou J. Yi T. Qian Y. Du X. (2018). Expression of tissue factor in human cervical carcinoma tissue. Exp. Ther. Med. 16 (5), 4075–4081. 10.3892/etm.2018.6723 30402151 PMC6200962

